# Reclassification of Glioblastoma Multiforme According to the 2021 World Health Organization Classification of Central Nervous System Tumors: A Single Institution Report and Practical Significance

**DOI:** 10.7759/cureus.21822

**Published:** 2022-02-01

**Authors:** George S Stoyanov, Emran Lyutfi, Reneta Georgieva, Radoslav Georgiev, Deyan L Dzhenkov, Lilyana Petkova, Borislav D Ivanov, Ara Kaprelyan, Peter Ghenev

**Affiliations:** 1 General and Clinical Pathology/Forensic Medicine and Deontology, Medical University of Varna, Varna, BGR; 2 Neurology and Neuroscience, Medical University of Varna, Varna, BGR; 3 Faculty of Medicine, Medical University of Varna, Varna, BGR; 4 Imaging Diagnostics, Interventional Radiology and Radiotherapy, Medical University of Varna, Varna, BGR; 5 Clinical Medical Sciences, Medical University of Varna, Varna, BGR

**Keywords:** demographics, who 2021, survival pattern, mgmt, idh, glioblastoma multiforme

## Abstract

Introduction

The 2021 World Health Organization (WHO) classification of tumors of the central nervous system (CNS) has introduced significant changes to tumor taxonomy. One of the most significant changes in the isolation of isocitrate dehydrogenase (IDH) mutant forms of glioblastoma multiforme (GBM) into separate entities, as well as no longer allowing for entries to be classified as not otherwise specified (NOS). As a result, this entity now includes only the most aggressive adult-type tumors. As such, established prognostic factors no longer apply, as they now form the criteria of different disease entries or have been established based on a mixed cohort. Herein, we aimed to reclassify glioblastoma cases diagnosed per the 2016 WHO tumors of the CNS classification into the 2021 WHO tumors of the CNS classification and establish a patient survival pattern based on age, gender, tumor location, and size as well as tumor O‐6‐methylguanine‐DNA methyltransferase (MGMT) mutation.

Materials and methods

A retrospective, non-clinical approach was utilized. Biopsy specimens of adults diagnosed with GBM, WHO grade 4, NOS in the period February 2018-February 2021 were reevaluated. The data regarding the patient's gender and age were withdrawn from the medical documentation. Immunohistochemistry was performed with mouse monoclonal anti-IDH R132H and rabbit polyclonal anti-MGMT. Radiology data on tumor location and size were pulled from the radiology repository. Data were statistically analyzed for significance, using Kaplan-Meier survival analysis, with a 95% confidence interval and p<0.05 defined as significant.

Results

A total of 58 cases fit the set criteria, with eight of them (13.7%) harboring an IDH R132H mutation and were hence reclassified as diffuse astrocytoma IDH-mutant, WHO CNS grade 4. The cases that retained their GBM classification included n=28 males and n=22 females, a male to female ratio of 1.27:1, and a mean age of 65.3 years (range 43-86 years). The MGMT mutational status revealed a total of n=17 positive cases (35%), while the remaining cases were negative. No hemispheric predilection could be established. Lobar predilection was as follows: temporal (37.78%), parietal (28.89%), frontal (24.44%), and occipital (8.89%).

The mean tumor size measured on neuroradiology across the cohort was 50.51 mm (range 20-76 mm). The median survival across cases was 255.96 days (8.41 months), with a range of 18-1150 days (0.59-37.78 months). No statistical correlation could be established between patient survival and gender, hemispheric location, lobar location, and tumor size. A significant difference in survival was established only when comparing the 41-50 age groups to the 71-80 and 81-90 age groups and MGMT positive versus negative tumors (p=0.0001).

Conclusion

From a practical standpoint, the changes implemented in the new classification of CNS tumors define GBM as the most aggressive adult type of tumor. Based on their significantly more favorable prognosis, the reclassification of IDH mutant forms of astrocytomas has had little epidemiological impact on this relatively common malignancy but has significantly underlined the dismal prognosis. The changes have also led to MGMT promoter methylation status being the only significant prognostic factor for patient survival in clinical use, based on its prediction for response to temozolomide therapy in this nosological unit clinically presenting when it has already reached immense size.

## Introduction

The 2021 World Health Organization (WHO) classification of tumors of the central nervous system (CNS) introduced significant changes to the tumor entities [1]. Chief among these are the criteria for the placement of the diagnosis of glioblastoma multiforme (GBM) [1,2]. While classically, these requirements were purely histological, based on the presence of pathognomonic features such as glomeruloid neovascular proliferation and pseudopalisadic necrosis (primary Scherer figures), now the diagnosis requires the lack of isocitrate dehydrogenase 1 and 2 mutations (IDH-wildtype) as well as a lack of mutation in histone 3 (H3-wildtype) [2]. These changes were introduced primarily to specify the prognosis of the diagnosis, as patients with this morphology and lack of mutations have a poor overall prognosis when compared to other tumors with similar morphology with the presence of the mutations mentioned above, as well as to underline the specificity of this tumor entry for the adult population. As such, the 2021 classification no longer allows the use of not otherwise specified (NOS) when reporting GBM, as well as introducing a new entry-astrocytoma, WHO CNS grade 4, consisting of the IDH mutant forms of tumors with identical morphology and diffuse hemispheric glioma, WHO CNS grade 4 for tumors with H3 G34 mutation [1-4].

The aim of this study was to reevaluate cases of GBM, reported as per the guidelines of the WHO 2016 classification of CNS tumors as NOS, and establish their survival pattern based on clinical and neuroradiologic features and their O‐6‐methylguanine‐DNA methyltransferase (MGMT) mutational status based on the new guidelines.

## Materials and methods

A retrospective, non-clinical approach was utilized. Biopsy specimens of adults diagnosed with GBM, WHO grade 4, NOS in the period February 2018-February 2021 were withdrawn from the pathology repository of a single tertiary healthcare institution. Cases were reevaluated for specimen morphology and remaining paraffin-embedded material (PEM) for immunohistochemical staining. Cases with insufficient remaining PEM and repeat biopsies in the period were excluded from the analysis.

A total of 58 cases fit the established criteria. The data regarding the patient's gender and age were withdrawn from the medical documentation. Radiology data on tumor location and size were pulled from the radiology repository. Data were not included for patients in which primary neuroradiology was performed in an outpatient setting. Immunohistochemistry (IHC) was performed with mouse monoclonal anti-IDH R132H, dilution 1:700 (catalog number MABC171, MilliporeSigma-Merck, Burlington, MA, USA). Immunohistochemistry for MGMT promoter methylation was performed with rabbit polyclonal anti-MGMT, dilution 1:700 (catalog number HPA069497, MilliporeSigma-Merck, Burlington, MA, USA). Immunohistochemistry was performed on a Dako autostainer link 48 (Agilent Technologies, Inc., Santa Clara, CA, USA) using the preprogrammed staining protocols, and the EnVision™ FLEX Mini Kit, high pH visualizing system (catalog number K8024, Agilent Technologies, Inc., Santa Clara, CA, USA).

Patient demographics and MGMT profile were statistically analyzed for significance, using Kaplan-Meier survival analysis, with a 95% confidence interval and p<0.05 defined as significant, calculated with MedCalc version 20.023 (MedCalc Software Ltd, Ostend, Belgium).

## Results

IDH mutation status

**Figure 1:**
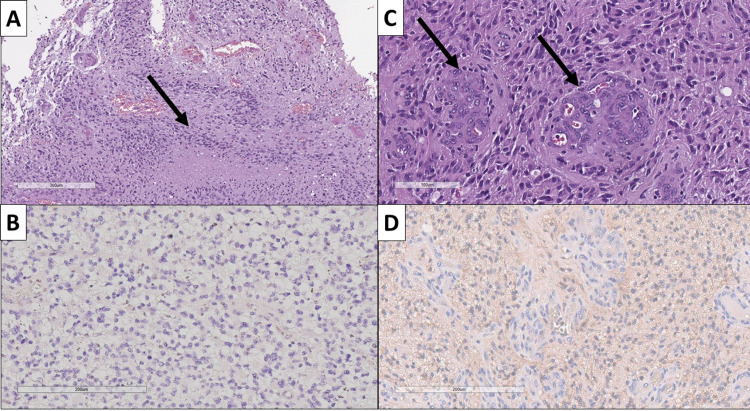
Morphological and immunohistochemical comparison of glioblastoma multiforme, WHO CNS grade 4, IDH wildtype and diffuse astrocytoma, WHO CNS grade 4, IDH mutant. (A) Glioblastoma multiforme, WHO CNS grade 4 with pseudopalisadic necrosis (arrow), H&E stain, original magnification ×80; (B) same tumor from (A), immunohistochemically negative for IDH R132H mutation, original magnification ×200; (C) diffuse astrocytoma, WHO CNS grade 4 with glomeruloid neovascular proliferation (arrows), H&E stain, original magnification ×200; (D) same tumor from (C), immunohistochemically positive for IDH R132H mutation, original magnification ×200. WHO: World Health Organization; CNS: central nervous system; H&E: hematoxylin and eosin; IDH R132H: isocitrate dehydrogenase mutation variant.

From the total of 58 selected cases that fit the selection criteria, a total of eight tumors (13.7%) harbored the IDH R132H mutation and were hence reclassified as diffuse astrocytoma IDH-mutant, WHO CNS grade 4 (Figure 1). The remainder of the tumors did not harbor the mutation and kept their classification as GBM.

Demographic characteristics of GBM, WHO CNS grade 4

Male (n=28) to female (n=22) ratio in GBM cases was 1.27:1, with a mean age of 65.3 years (range 43-86 years), with four cases diagnosed in the age group 41-50 (all males), 14 cases in the age group 51-60 (nine males and five females), 17 cases in the age group 61-70 (nine males and eight females), 11 cases in the age group 71-80 (four males and seven females), and four cases in the age group 81-90 (two cases for males and females each).

MGMT mutation status

**Figure 2:**
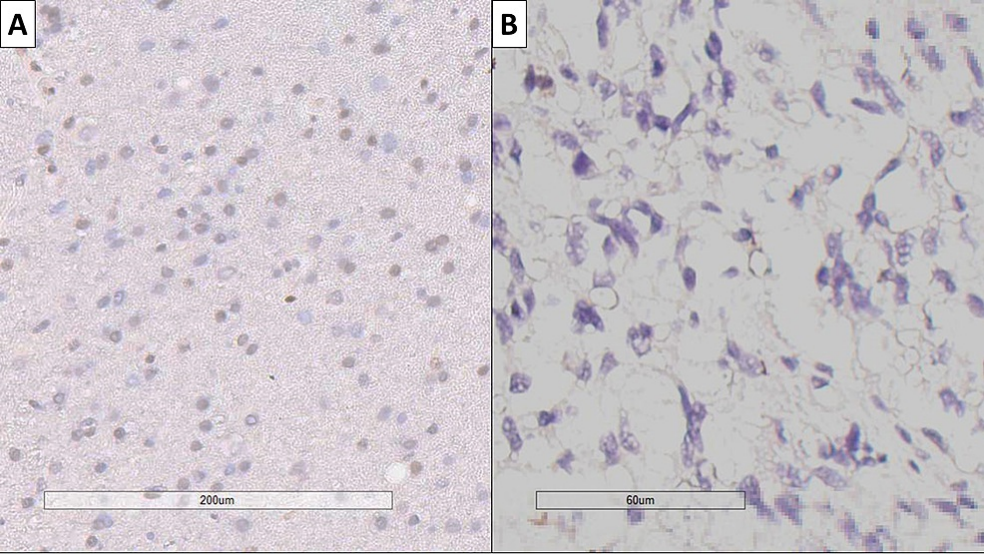
MGMT status in glioblastoma. (A) MGMT positive tumor, IHC marking for MGMT, original magnification ×200; (B) MGMT positive tumor, IHC marking for MGMT, original magnification ×400. MGMT: O‐6‐methylguanine‐DNA methyltransferase; IHC: immunohistochemistry.

As observed on immunohistochemistry, MGMT mutational status revealed a total of 17 positive cases (35%), while the remaining cases were negative (Figure 2). The mean age of MGMT positive cases was 62.42 years (range 43-77 years), whilst MGMT negative cases had a mean age of 66.79 years (range 50-86 years). Higher incidence of MGMT positive GBM was observed in females (n=9) compared to males (n=8), on the background of the higher GBM incidence in males.

Neuroradiology parameters

Hospital-based neuroradiology was available on 45 of the 50 cases that retained their GBM, WHO CNS grade 4 diagnosis. No hemispheric predilection could be established based on tumor location, as 25 tumors were located in the left cerebral hemisphere and the remaining 20 in the right one. A total of 18 tumors (40%) affected more than one lobe of the hemisphere, with one case (2.22%) presenting as a multicentric glioma. Lobar predilection, based on the lobe with the most extensive involvement in cases of more than one lobe being affected, favored the temporal lobe with 17 cases (37.78%), followed by parietal (n=13, 28.89%), frontal (n=11, 24.44%), and occipital (n=4, 8.89%).

Mean tumor size measured on neuroradiology across the cohort was 50.51 mm, median 51 mm, mode 45 mm, and range 20-76 mm. Frontal lobe tumors had a mean size of 52.55 mm (range 30-67 mm), temporal lobe tumors had a mean size of 57.56 mm (range 40-76 mm), parietal lobe tumors had a mean size of 42.77 mm (range 21-76 mm), and occipital lobe tumors had a mean size of 39.75 mm (range 20-51 mm). No significant difference was observed in lobar tumor size when comparing left-sided to right-sided tumors in the same lobe, except for parietal lobe tumors, where the mean tumor size of left-sided tumors was 39.38 mm (range 21-76 mm), compared to the mean size of 48.2 mm (range 31-60) for right-sided tumors.

Survival

Of the 50 cases included in the study, 45 patients had expired, whilst five were still alive. The median survival across cases was 255.96 days (8.41 months) with a range of 18-1150 days (0.59-37.78 months), with a slightly higher survival observed in males (mean 274.43 days, range 23-1150) compared to females (232.46 days, range 18-1061). One-year survival rates were 26% (n=13), two-year survival rates were 8% (n=4), and three-year survival rates were 4% (n=2).

Statistical analysis

**Figure 3:**
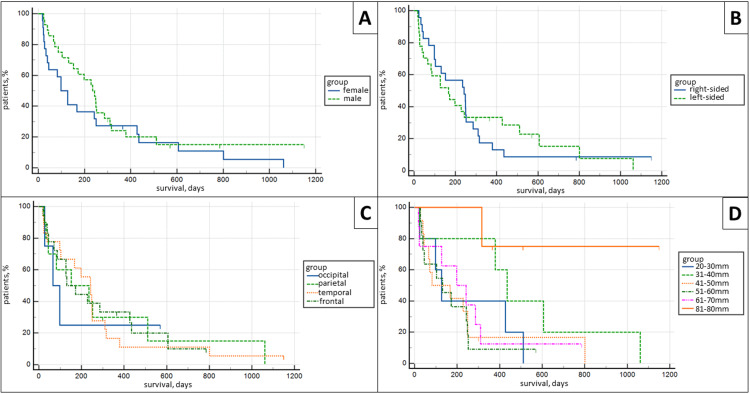
Kaplan-Meier survival analysis curves. (A) Survival comparison between females and males (p>0.05); (B) survival comparison between left-sided and right-sided tumors (p>0.05); (C) survival comparison between location in lobe (p>0.05); (D) survival comparison between tumor sizes (p>0.05).

Kaplan-Meier survival analysis revealed no statistical difference between patient survival and gender, survival and hemispheric location (left and right hemisphere), survival and lobar location (frontal, parietal, temporal, and occipital), as well as survival and tumor size, when tumors were separated into 20-30 mm, 31-40 mm, 41-50 mm, 51-60 mm, 61-70 mm, and 71-80 mm groups, p<0.05 (Figure 3).

**Figure 4:**
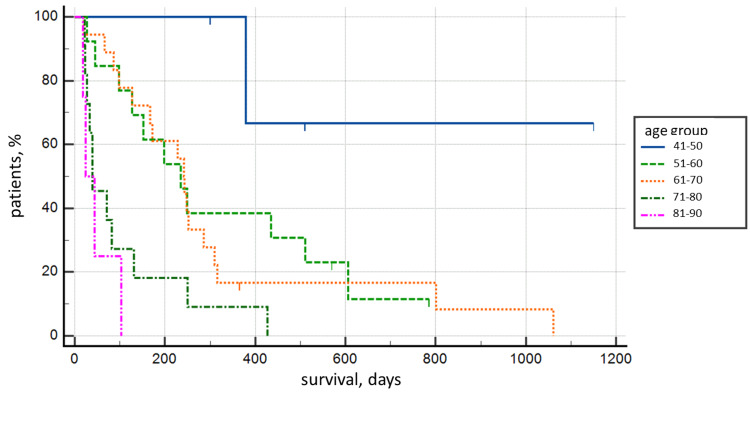
Kaplan-Meier survival analysis curve between age groups (p=0.0059).

Statistical correlation for patient survival was established only for patient age when the 41-50 year old age group was compared with the 71-80 year old and 81-90 year old age groups, both on Kaplan-Meier survival curves (p=0.0059) and post-comparison Tukey test, p<0.05 (Figure 4 and Table 1).

**Table 1:** Post-comparison Tukey test for survival comparison.

Survival compared to the 41-50 age group	Difference in mean survival	df	p<0.05
51-60 Age group	278.32	2.97	No
61-70 Age group	300.28	3.27	No
71-80 Age group	480.75	4.98	Yes
81-90 Age group	537.5	4.6	Yes

**Figure 5:**
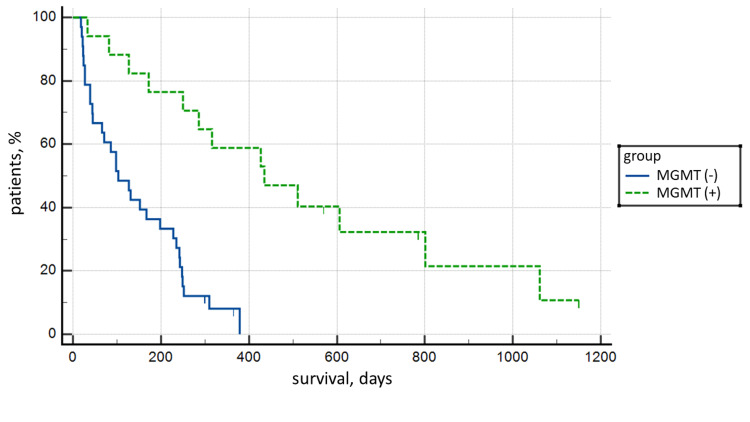
Kaplan-Meier survival analysis curve between MGMT positive and negative tumors (p<0.0001). MGMT: O‐6‐methylguanine‐DNA methyltransferase.

The highest statistical significance of Kaplan-Meier survival curves was established based on MGMT mutation status: the mean survival of MGMT positive cases was 477.77 days (15.7 months) compared to 141.58 days (4.65 months) for MGMT negative cases, with p<0.0001 (Figure 5).

## Discussion

The changes in the new WHO classification of CNS tumors from 2021, although at first glance drastic compared to the previous classifications, are primarily based on clinically significant and detectable changes in the phenotype of tumors [1]. Given their smooth implementation with the consortium to inform molecular and practical approaches to CNS tumor taxonomy (cIMPACT-NOW) reports, giving not only timely information to practicing neuropathologists but also time to adapt the diagnostic process before the official introduction of changes [2]. Furthermore, the cIMPACT-NOW reports allow for timely infrastructure and resource provision of the process. As such, the adaptation to the new classification should not be complex for many of the developed neuropathological centers [2]. Finally, a large part of the innovations concern either previously introduced nosological units with a change in the name or rare subforms of tumors with a well-clarified diagnostic approach to them.

Concerning GBM and the new diffuse astrocytoma entry, IDH mutant, WHO CNS grade 4, given the partial presence of IDH in the previous revision and the well-known frequency and significance of the mutation, this change is cosmetic rather than functional and in large does not change the diagnostic approach [5]. The changes in lower-grade astroglial tumors are more drastic with the introduction of molecular criteria for a grade, which is why in the new classification it should be noted as a WHO CNS grade, given the integrated way of its placement. The change with the introduction of the pediatric group of diffuse gliomas, some of which have so far belonged to the group of GBM, is a cosmetic modification rather than a functional one, and this should not be a diagnostic difficulty, given the rapid adaptation to midline gliomas, which also have H3 mutations, introduced in the previous classification [1].

In our cohort, as already mentioned, an IDH mutant phenotype was found in 13.7% (n=8) of a total of 58 patients in the primary cohort, allowing reclassification of tumors according to the new classification. In this aspect, our data is fully comparable to other extensive studies where the frequency of IDH R132H mutant forms of GBM, currently diffuse astrocytoma, WHO CNS grade 4, varies by about 10%, with other forms of mutation in IDH1 as well as mutations in IDH2 have been shown to be rare entries, rather than a statistically significant form of mutation [6]. The gender and age distribution are also comparable with these studies, as mutant forms are more common in younger individuals (under 50 years of age) and females [6].

In our cohort, other forms of mutations in IDH1 and IDH2 that would lead to a new reclassification of tumors have not been studied, not only because of their extreme rarity but also because of the small size of the cohort itself and its age composition, in which these mutant forms are sporadic [6].

Given the small number of cases reclassified, the incidence of GBM in our population, which is reported at 2.03 cases per 100,000 capita, will not drastically change. However, these figures are significantly smaller than those reported in the United States-3.23 cases per 100,000 capita, despite the comparable median age of 65 years, coupled with a significantly higher incidence in males-4.04, compared to 2.53 for females [7,8].

However, the reclassification of this small number of cases will lead to a drastic increase in the average age of onset of the disease, given that mutant forms are more common in young people under 50, as well as a further statistical increase in incidence for males due to the higher incidence of mutant forms in females [9]. For example, the current average age in our cohort is 65.3 years, while following the previous classification, we reported it to be 59.18 years [10]. Another direct nuance of the change in classification is the reduction in the average survival of the patients, given that patients with IDH mutant forms have significantly longer survival. Apparently, with these modifications to the classification, the disease definition of GBM can be summed up as a primary CNS tumor characteristic of the elderly, more common in males, and with a poor prognosis.

For this reason, Kim defines GBM from a clinical and epidemiological point of view as a neurological disease characteristic of older men [11]. In addition, Ostrom also reports an average annual incidence of 3.99 per 100,000 capita for males and a significantly lower 2.52 per 100,000 capita for females in a similar age-dependent pattern to the current study [12]. In the same study, Ostrom also performed a survival analysis. Although the analysis was based on the WHO classification for CNS tumors from 2016, a subanalysis of GBM with and separately without IDH mutation was performed. In the analysis of tumors without IDH mutations, corresponding to the new WHO definition of GBM, the median survival is 16.9 months, with the median survival for males being 15 months, compared to 25.5 months for females. Based on this epidemiological data, GBM is not only a more common tumor in males but also has a significantly worse prognosis in them.

However, as can be seen from our data, in our population, there is no significant difference in survival rates between males and females, and the average survival rate for males is even higher than that for females. It is also evident from the comparative analysis with the central brain tumor registry of the United States (CBTRUS) that in our cohort, the average survival is significantly lower-8.41 months compared to 16.9 months, i.e., more than double the difference [7]. However, the survival in the current cohort is an encouraging factor, given its sharp rise compared to previous studies in our population, in which even in the presence of an IDH mutant, the mean survival was 197.5 days (6.49 months) [10].

Despite our cohort's significantly lower average survival, the survival rates in the first year-26%, the second year-8%, and the third year-4% are comparable to those reported by other authors [13].

As seen from our sample and comparison with other cohorts, GBM is a tumor with an abysmal prognosis. Despite the vast resources devoted to studying the biological behavior of tumors and the potential for pharmacological and nonpharmacological interventions, historical survival has remained almost unchanged [10]. As can be seen from CBTRUS data, our samples, and limited data from the National Cancer Registry of the Republic of Bulgaria and the global cancer observatory (GLOBOCAN), GBM is the most common tumor of the nervous system and, according to some statistics, is more common than some malignant diseases with significantly higher public awareness rates, such as laryngeal carcinoma, thyroid malignancies, and Hodgkin's lymphoma [8,10,14]. Also evident from these large statistical samples is the higher incidence not only of GBM but also of all types of tumors of the CNS in developed countries, compared to developing ones [15]. This fact, of course, is mainly related to access to medical care and the existence of diagnostic modalities for these nosological units, and not due to actual lower incidence [16]. Unfortunately, the lower rate of morbidity found in our country compared to the countries with high economic resources puts us in the group of countries with a relatively high rate of hidden morbidity [7,8]. Given the relatively high incidence of GBM, attempts at early diagnosis programs have been reported in some regions, given the impossibility of prevention programs, such as the ones for malignancies of the uterine cervix, lung, larynx, colon, and stomach [17]. However, due to the lack of effective screening, unlike other malignancies such as prostate, colon, mammary, and thyroid malignancies and the high-cost neuroradiology evaluations, these programs are ineffective in the first place in terms of financial resources and, secondly, have little impact on the survival of patients [17]. Currently, the only cost-effective screening method, despite its low specificity, is the study of circulating glial fibrillary acidic protein (GFAP) levels, which is more indicative of the presence of a pathological process in the CNS and more effective in the follow-up of GBM patients due to elevated circulating levels also present in the serum of patients with other tumors, including metastatic, as well as in cerebrovascular diseases, inflammatory diseases, and neurodegenerative processes [18,19].

However, the primary factor in the survival of patients with GBM remains the presence of MGMT promoter methylation, which indicates a good response to temozolomide therapy. In our cohort, the incidence of MGMT-positive tumors is 35% (n=17), which is comparable to data reported by other cohorts, where the incidence varies between 30% and 60% [20,21]. In our cohort, the difference in survival was statistically significant at p=0.0001, with a mean survival in MGMT-positive tumors of 477.77 days versus 141.58 days in negative tumors, with statistical significance comparable to that reported in other cohorts. From this data, we can conclude that the significant difference in survival between our cohort and that reported in others is not due to a difference in survival of MGMT positive cases, where it is similar, but mainly to MGMT negative cases, where survival is significantly below the reported.

Concerning tumor localization, it is difficult in this transitional period of classification to compare with the literature data due to differences in the biological course of tumors with and without IDH mutation. One of the few studies on the localization of IDH mutant forms shows that they are most commonly detected in the frontal lobe [6].

Regarding the tumor localization, Flores in a cohort of 44 patients, including only cases of GBM according to the WHO classification from 2021 and very close to our demographic characteristics, found that the majority of tumors (59.1%) are located in the right cerebral hemisphere, and the most common localization relative to the lobe is the frontal (29.5%), followed by temporal and parietal with 25%, and the rarest localization in the occipital lobe [22]. In another study, similar in volume and gender distribution but with a significantly lower mean age (49.05 years), Abd-Elghany found that again the most common site of GBM was in the frontal lobe, followed by the temporal, parietal, occipital lobes, and most rarely-subtentorial [23]. Despite the differences in localization between the three cohorts, the most common localization is temporal, followed by parietal, frontal, and occipital, underlining the diffuse origin of the tumor from different brain structures.

A relatively large sample of gliomas, including n=116 GBM according to the old WHO criteria Larjavaara, found that gliomas are most often located in the frontal lobe, followed by the temporal, parietal, and are rarely occipital [24]. In the same study, the smallest tumor volume was established in the occipital lobe, followed by the parietal, with the largest volume in the temporal lobe with a negligible difference from the frontal [24]. Here again, despite the difference in histogenetic criteria and the difference in the distribution of tumors relative to the lobes of the CNS, the results in terms of neuroradiologically determining tumor size in the patient presentation are entirely comparable. The reported smaller size in the occipital and parietal lobes is related to the earlier onset of symptoms. These are predominantly due to the involvement of critical systems-visual, somatosensory, and somatomotor. In our cohort, the tumor size of parietal tumors on the left side was not only significantly smaller when compared to other locations, excluding occipital ones, but also smaller than that of right-sided parietal tumors. This would directly correlate to earlier motor symptoms in right-handed people with a tumor in this location than symptom onset in right-sided tumors.

The IDH mutant form is a major part of the secondary GBM group (diffuse astrocytoma, WHO CNS grade 4 as per the 2021 guidelines)-originating on the basis of a previous low-grade glioma. These forms of astrocytoma are, by definition, slow-growing, less aggressive, and clinically present in smaller sizes while still being aggressive tumors. In a mixed cohort (tumors with and without IDH mutation), Stensjoen, through repeated neuroradiological studies in a total of 106 patients before surgery, found that the daily rate of tumor volume increase was 1.4%, with a time to doubling tumor volume of 49.6 days [25]. In a smaller cohort (n=32), with the same characteristics and using similar methods, Wang determined an even faster time to doubling of the tumor volume of 17 days [26]. In a case report of a 60-year-old man, Zhang neurologically illustrated the progression of GBM from a 7 mm lesion to a 13 mm lesion on day 12, 17 mm on day 23, and involvement of almost the entire hemisphere seven months after patient presentation, estimating a time to tumor size doubling of about ten days [27]. If we assume that 10% of the tumors in these cohorts are IDH mutants, as per the 2021 WHO criteria, these figures for GBM will undoubtedly show even more aggressive growth.

Given the rapid growth of the tumor and its diffuse nature, which cannot be well emphasized neuroradiologically, neurosurgical interventions in GBM are difficult methodologically and with a consequent neurological deficit, but affect patient survival only in extensive resection. In an analysis of the survival of more than 400 patients with GBM according to the old classifications, Lacroix found that only excision of more than 85% of the neuroradiologically determined tumor volume showed improvement in patient survival compared to biopsy and limited resection, with a statistically significant difference in survival observed on resection of more than 98% of the neuroradiologically established tumor volume [28].

Study limitations

The first limitation of our study is the small cohort gathered in the specified time interval and the short follow-up of patients-a little over three and a half years. Thus, although small and probably without statistical significance, the final survival and its variables, which vary according to the various factors studied, will have an absolute value that differs, albeit to a small extent, from those derived so far. Furthermore, as preoperative neuroradiology was not available for all patients, the neuroradiology analysis does not fully represent the complete parameters of all cases studied.

Another major drawback is the lack of study of other rare noncanonical forms of IDH mutations, such as IDH2 mutations and unconventional mutations in IDH1 other than R132H and the establishment of H3 mutation status. These alterations themselves, compared to the new WHO criteria for classifying CNS tumors from 2021, would reclassify tumors with these alterations to high-grade diffuse astrocytoma, WHO CNS grade 4; hemispheric glioma, WHO CNS grade 4, and midline glioma (pontine glioma), WHO CNS grade 4. Given the location of the tumors described in the cohort, as well as the age group of patients and the relatively low incidence of tumors with the described mutations in the general population, the presence of these tumor groups is unlikely and should not affect the significance of the statistically derived results.

## Conclusions

From a practical standpoint, the changes implemented in the 2021 WHO classification of CNS tumors more strictly define GBM as a highly aggressive tumor with an unfavorable outcome. The reclassification of IDH mutant forms of astrocytomas with morphology identical to that of GBM, based on their significantly more favorable prognosis, has had little epidemiological impact on this relatively common malignancy. However, these changes have more strictly defined the biological behavior of GBM as an aggressive tumor, clinically presenting with immense size. The changes have also led to MGMT promoter methylation status being the only significant prognostic factor for patient survival in clinical use, based on its prediction for response to temozolomide therapy.
